# Expanding the Repertoire of Carbapenem-Hydrolyzing Metallo-ß-Lactamases by Functional Metagenomic Analysis of Soil Microbiota

**DOI:** 10.3389/fmicb.2016.01985

**Published:** 2016-12-26

**Authors:** Dereje D. Gudeta, Valeria Bortolaia, Simona Pollini, Jean-Denis Docquier, Gian M. Rossolini, Gregory C. A. Amos, Elizabeth M. H. Wellington, Luca Guardabassi

**Affiliations:** ^1^Department of Veterinary Disease Biology, Faculty of Health and Medical Sciences, University of CopenhagenFrederiksberg, Denmark; ^2^Department of Medical Biotechnology, University of SienaSiena, Italy; ^3^Department of Experimental and Clinical Medicine, University of FlorenceFlorence, Italy; ^4^Clinical Microbiology and Virology Unit, Florence Careggi University HospitalFlorence, Italy; ^5^Don Carlo Gnocchi FoundationFlorence, Italy; ^6^School of Life Sciences, University of WarwickCoventry, UK; ^7^Department of Biomedical Sciences, Ross University School of Veterinary MedicineSt. Kitts, West Indies

**Keywords:** carbapenems, antibiotic resistance, soil, functional metagenomics, metallo-ß-lactamases

## Abstract

Carbapenemases are bacterial enzymes that hydrolyze carbapenems, a group of last-resort β-lactam antibiotics used for treatment of severe bacterial infections. They belong to three β-lactamase classes based amino acid sequence (A, B, and D). The aim of this study was to elucidate occurrence, diversity and functionality of carbapenemase-encoding genes in soil microbiota by functional metagenomics. Ten plasmid libraries were generated by cloning metagenomic DNA from agricultural (*n* = 6) and grassland (*n* = 4) soil into *Escherichia coli*. The libraries were cultured on amoxicillin-containing agar and up to 100 colonies per library were screened for carbapenemase production by CarbaNP test. Presumptive carbapenemases were characterized with regard to DNA sequence, minimum inhibitory concentration (MIC) of β-lactams, and imipenem hydrolysis. Nine distinct class B carbapenemases, also known as metallo-beta-lactamases (MBLs), were identified in six soil samples, including two subclass B1 (GRD23-1 and SPN79-1) and seven subclass B3 (CRD3-1, PEDO-1, GRD33-1, ESP-2, ALG6-1, ALG11-1, and DHT2-1). Except PEDO-1 and ESP-2, these enzymes were distantly related to any previously described MBLs (33 to 59% identity). RAIphy analysis indicated that six enzymes (CRD3-1, GRD23-1, DHT2-1, SPN79-1, ALG6-1, and ALG11-1) originated from *Proteobacteria*, two (PEDO-1 and ESP-2) from *Bacteroidetes* and one (GRD33-1) from *Gemmatimonadetes*. All MBLs detected in soil microbiota were functional when expressed in *E. coli*, resulting in detectable imipenem-hydrolyzing activity and significantly increased MICs of clinically relevant ß-lactams. Interestingly, the MBLs yielded by functional metagenomics generally differed from those detected in the same soil samples by antibiotic selective culture, showing that the two approaches targeted different subpopulations in soil microbiota.

## Introduction

ß-lactams were introduced in clinical practice in the 1940s and currently account for more than half of all antibiotic prescriptions for human use (Tahlan and Jensen, 2013). Among the β-lactams, carbapenems are last resort drugs for treatment of life-threatening nosocomial infections caused by multidrug-resistant Gram-negative pathogens. Carbapenem resistance mediated by the production of carbapenem-hydrolizing enzymes (carbapenemases) has spread globally in Enterobacteriaceae (Nordmann et al., 2011). Carbapenemases are classified into classes A, B, and D according to the Ambler classification of β-lactamases based on amino acid sequence similarity (Ambler, 1980). Classes A and D comprise serine-utilizing enzymes, whereas enzymes belonging to class B, also called metallo-ß-lactamases (MBLs), require zinc as an essential co-factor (Walsh et al., 2005). MBLs are inhibited by metal chelators such as EDTA and are not susceptible to the currently available ß-lactam inhibitors such as clavulanate, sulbactam, tazobactam, and avibactam (Walsh et al., 2005; Cornaglia et al., 2011). Based on sequence similarity, MBLs are classified into subclasses B1, B2, and B3 (Galleni et al., 2001). Subclass B1 comprises most of the MBLs acquired by bacteria of clinical relevance, such as IMP, VIM, and NDM (Nordmann et al., 2011). Subclasses B2 and B3 have mostly been described in environmental bacteria as resident (i.e., not associated with mobile genetic elements) resistance determinants (Rossolini et al., 2001; Saavedra et al., 2003; Stoczko et al., 2006). The only exceptions are AIM-1 and SMB-1, which have been reported in clinical isolates of *Pseudomonas aeruginosa* and *Serratia marcescens*, respectively (Wachino et al., 2011; Yong et al., 2012). B1 and B3 enzymes generally exhibit broad β-lactam substrate profiles, whereas B2 enzymes preferentially hydrolyze carbapenems (Bebrone et al., 2009; Fonseca et al., 2011).

The origins of the carbapenemases found in clinically relevant bacteria are unknown. Soil bacteria represent an important reservoir of antibiotic resistance determinants, including β-lactamases (Allen et al., 2009; Forsberg et al., 2012; Nesme and Simonet, 2015), and therefore a possible reservoir of carbapenemases in nature. However, little information is available about the occurrence and types of carbapenemases in soil microbiota. In a previous study, we have shown that subclass B3 MBLs are widespread in different genera of the culturable fraction of soil bacteria (Gudeta et al., 2016a). Since only a small fraction of the soil microbiota is culturable, the actual diversity of carbapenemases is likely greater than what it could be appreciated by culture-dependent methods. The aim of this study was to elucidate occurrence, diversity, and functionality of carbapenem-hydrolyzing enzymes in soil microbiota using a functional metagenomics approach. We identified new types of carbapenemases, demonstrated their functionality in *Escherichia coli*, and predicted the most likely bacterial taxa from which they originated.

## Materials and methods

### Soil samples

Ten soil samples were selected from a collection of 25 samples that were previously screened for the occurrence of carbapenemase-producing bacteria by antibiotic selective culture (Gudeta et al., 2016a). We selected one sample per soil type and per geographical origin (Table 1). If more than one soil sample met these criteria, we selected a sample not yielding any carbapenemase-producing strains by selective culture.

**Table 1:** **The 10 soil samples and corresponding yields of genes encoding metallo-β-lactamases obtained by functional metagenomics (this study) and by antibiotic selective culture (Gudeta et al., 2016a)**.

**Sampling site**	**Soil type**	**Soil ID^a^**	**Library**	**Yield by functional metagenomics**						**Yield by selective culture**		
			**coverage (Gb)**									
				**Total clones**	**Clones screened by CarbaNP**	**CarbaNP-pos. clones**	**Clones with unique insert**	**Annotated MBL gene**	**Subclass**	**MBL producers**	**Annotated MBL gene**	**Subclass**
Amagerfælled, Denmak	Grassland	1	1.2	>200	100	7	2	*bla*_GRD23−1_	B1	0	−	−
								*bla*_GRD33−1_	B3			
Lungaard, Denmark	Agricultural	6	2.4	>200	100	0	0	−	−	0	−	−
Højgaard, Denmark	Agricultural	7	0.75	>500	100	0	0	−	−	0	−	−
Hygum, Denmark	Grassland	4	1.7	>200	100	0	0	−	−	1	*bla*_THIN−B_	B3
Copenhagen, Denmark	Agricultural	3	1.4	>500	100	1	1	*bla*_CRD3−1_	B3	1	*bla*_L1_	B3
Dossenhem, Germany	Agricultural	10	2	>200	100	0	0	_−_	_−_	0	_−_	_−_
Dossenhem, Germany	Agricultural	11	2.5	>200	100	48	1	*bla*_DHT2−1_	B3	0	−	−
Djelfa, Algeria	Grassland	12	1.5	17	17	15	2	*bla*_ALG6−1_	B3	0	−	−
								*bla*_ALG11−1_	B3			
Abanilla, Spain	Agricultural	21	1.6	98	98	14	1	*bla*_SPN79−1_	B1	0	−	−
Svalbard, Norway	Grassland	23	0.5	75	75	9	2	*bla*_PEDO−1_	B3	4	*bla*_SPG−1_	B3
								*bla*_ESP−2_	B3		*bla*_PEDO−2_	B3
											*bla*_THIN−B_	B3
											*bla*_L1_	B3

a *Soil ID number according to Gudeta et al. (2016a)*.

### Metagenomic library construction and screening

Metagenomic DNA was purified from one gram of soil by PowerSoil® DNA Isolation Kit (MO BIO Laboratories, CA, USA) using four power-bead tubes per sample. After verifying the quality of the DNA yield by gel electrophoresis, DNA was sheared by Ultrasonic homogenizer (BANDELIN electronic GmbH & Co. KG) to approximately 0.5–7 kb fragments as previously described (Gudeta et al., 2016a). Fragments with size ranging 1.5–5 kb were separated by electrophoresis and purified by GeneJET Gel Extraction Kit (Thermo Scientific, Vilnius, Lithuania). Gel-purified DNA was blunted in a standard 50-μl reaction by End-It™ DNA End-Repair Kit (Epicentre, Madison, USA). Blunted DNA was purified and ligated into HincII-linearized pZE21MCS vector as previously described (Gudeta et al., 2016a). The ligation mix was dialyzed for 2 h using 0.025 μm DNA filter paper (Millipore, Massachusetts, USA) and distilled water. A 4-μl aliquot of the ligation mix was used to transform One Shot® TOP10 Electrocomp™ *Escherichia coli* (Invitrogen, Carlsbad, USA) using Gene Pulser II (Bio-Rad, California, USA). One milliliter of SOC medium was added to the transformation mix and incubated at 37°C with shaking. After 1 h incubation, 400 μl were plated on Luria Bertani (LB) agar (Difco, Le Pont-de-Claix, France) containing 50 μg/ml of kanamycin and 30 μg/ml of amoxicillin (Sigma-Aldrich, Steinheim, Germany). Library coverage was estimated by plating a ten-fold diluted suspension of the recovered cells on LB agar containing 50 μg/ml of kanamycin according to the procedure described by Sommer et al. (2009). The remaining library was enriched overnight in LB broth containing 50 μg/ml of kanamycin followed by subculture on LB agar (Difco, Le Pont-de-Claix, France) containing 50 μg/ml of kanamycin and 30 μg/ml of amoxicillin. Amoxicillin was used for initial screening to facilitate detection of carbapenemases, as previously described by various authors (Poirel et al., 2000; Bellais et al., 2002; Girlich et al., 2010).

### Detection of *E. coli* recombinant clones expressing carbapenemase

Up to 100 randomly selected amoxicillin-resistant colonies per sample were screened for carbapenemase production by CarbaNP test (Dortet et al., 2014). Briefly, cells were lysed in 100 μl of Tris-HCl buffer (Thermo Scientific, Rockford, Il, USA) and the lysate was mixed with 100 μl of phenol red solution containing 6 mg/ml imipenem/cilastatin (Fresenius Kabi, Bad Homburg, Germany). Phenol red solution without imipenem was included in the test as a negative control. After incubating at 37°C for a maximum of 2 h, red to orange/yellow color shift in the test vial and no color change in the negative control were interpreted as imipenem hydrolysis. Plasmid inserts of the carbapenemase-producing clones were sequenced using the primers described in Table 2. Sequences displaying less than 70% amino acid sequence identity to known MBLs were defined as new MBLs, as suggested by Cornaglia et al. (2007).

**Table 2:** **Primers used in this study**.

**Primer**	**Sequences (5′–3′)**	**Description**
HinCIIF	CCCCCCCTCGAGGTC	Forward primer targeting the flanking region of the cloning site in pZE21MCS
HinCIIR	ATCAAGCTTATCGATACCGTC	Reverse primer targeting the flanking region of the cloning site in pZE21MCS
GRD23F	CACTCGCCGCAGCCAGCTCCT	Internal forward primer for sequencing the DNA insert harboring *bla*_GRD23−1_
CRD3F	CAGTCGCTCGTCGAGGCGCCGG	Internal forward primer for sequencing the DNA insert harboring *bla*_CRD3−1_
CRD3R	CGATCGGGCGAGGCGTCTTG	Internal reverse primer for sequencing the DNA insert harboring *bla*_CRD3−1_
SPN79F	GGCGGCTGCTTCGTGAAGGG	Internal forward primer for sequencing the DNA insert harboring *bla*_SPN79−1_
SPN79R	CGCCGGCGCGGGCGCGCACG	Internal reverse primer for sequencing the DNA insert harboring *bla*_SPN79−1_
DHT2F	GACCATGCCGGCCCCGTCGCGA	Internal forward primer for sequencing the DNA insert harboring *bla*_DHT2−1_
DHT2R	AGGCGAACGCGGCTGCAAGA	Internal reverse primer for sequencing the DNA fragment harboring *bla*_DHT2−1_
ALG6F	GCGACCGTGGCGGCGAGCATC	Internal forward primer for sequencing the DNA insert harboring *bla*_ALG6−1_
ALG6R	GCGCACGGGATGCCGTGGCTC	Internal reverse primer for sequencing the DNA insert harboring *bla*_ALG6−1_
ALG11F	CACGGTGGCCGCGCTGCCGTG	Internal forward primer for sequencing the DNA insert harboring *bla*_ALG11−1_
ALG11R	CCGCGCCAGCAGGTCTCTGGC	Internal reverse primer for sequencing the DNA insert harboring *bla*_ALG11−1_
OSN5R	CCTACCGATACGCCAAAAGAG	Internal reverse primer for sequencing the DNA insert harboring *bla*_PEDO−1_

### Determination of minimum inhibitory concentration (MIC) and carbapenemase activity

The MICs of selected ß-lactams were measured in carbapenemase-producing recombinant *E. coli* TOP10-derived clones by broth microdilution using Sensititre ESBL plates (Trek Diagnostic Systems, OH, USA). The MICs of third-generation cephalosporins, cefepime, imipenem, and meropenem that fell outside the range of concentrations included in these commercial plates were determined by the broth microdilution method according to the Clinical Laboratory Standards Institute (CLSI) guidelines (Clinical Laboratory Standards Institute, 2015). Carbapenemase activity in bacterial crude extracts was determined by UV spectrophotometry as described previously (Lauretti et al., 1999), using 150 μM imipenem as the substrate in a Cary 100 UV-Vis spectrophotometer (Varian, Walnut Creek, CA).

### Bioinformatic analysis

The sequences of carbapenemase-encoding genes were used as queries in BLASTX in the NCBI database (default parameters). Hits showing maximum identity to the query sequence, except putative homologous proteins with unknown function, were downloaded into a local database for sequence alignment and amino acid comparison. MBLs with previously determined 3-D structure were used for structural alignments to ascertain if the known metal-binding amino acids were conserved in the new MBLs detected in this study. Additional sequences of previously described MBLs were obtained from published studies and added to the local database for phylogenetic tree construction. Amino acid sequence alignment was performed by MUSCLE (http://www.phylogeny.fr/one_task.cgi?task_type=muscle). Maximum likelihood analysis was performed by raxmlGUI 1.5b (Silvestro and Michalak, 2012) using the WAG amino acid substitution model, which was selected using Akaike Information Criterion implemented in PROTTEST 3 (Darriba et al., 2011). The data were analyzed using rapid bootstrap algorithm with 1000 bootstrap replicates. The phylogenetic tree was visualized by FigTree v1.4.0 (http://tree.bio.ed.ac.uk/software/figtree/) and the possible bacterial hosts of MBL-encoding genes were predicted by RAIphy based on comparison of relative abundance of unique 7-mers in the query sequence with reference genomes (Nalbantoglu et al., 2011; Forsberg et al., 2014).

### Accession numbers

The nine MBL nucleotide sequences described in this study have been submitted to GenBank and assigned accession numbers KU167035 to KU167043.

## Results

### Library coverage and carbapenemase activity of recombinant clones

The 10 constructed plasmid libraries, each containing DNA fragments of 1.5 kb on average, collectively covered an estimated size of 16 Gb, corresponding to over 3500 *E. coli* genome equivalents. Only three libraries yielded amoxicillin-resistant colonies after direct plating onto LB agar containing kanamycin and amoxicillin (3–84 colonies per library), whereas all libraries yielded amoxicillin-resistant colonies (17 to > 500 colonies per library) when plating onto selective agar was preceded by overnight enrichment in LB containing kanamycin, proving the usefulness of this enrichment step for detection of amoxicillin-resistant recombinant clones. Carbapenemase-producing recombinant clones were detected by CarbaNP in six of the 10 libraries (Table 1). The overall rate of carbapenemase-positive colonies was 10.6% (94/890).

### Identification and phylogeny of carbapenemases detected by functional metagenomics

**Figure 1:**
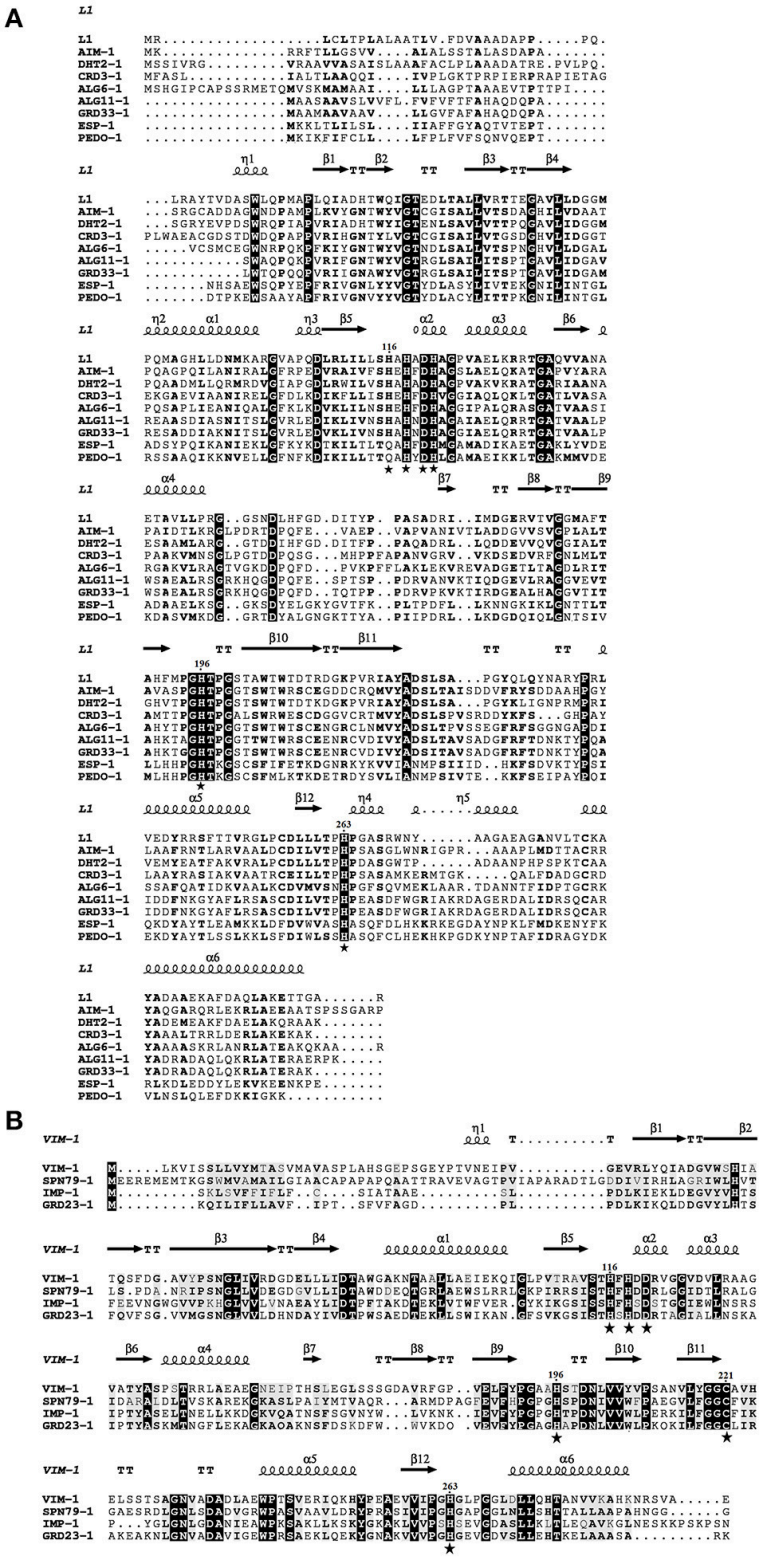
**Amino acid alignment of the new subclass B3 (A) and B1 (B) metallo-ß-lactamases (MBLs) detected in this study with known MBLs whose 3-D structure has been resolved by X-ray crystallography**. Zinc-binding amino acids are indicated by a star.

**Figure 2:**
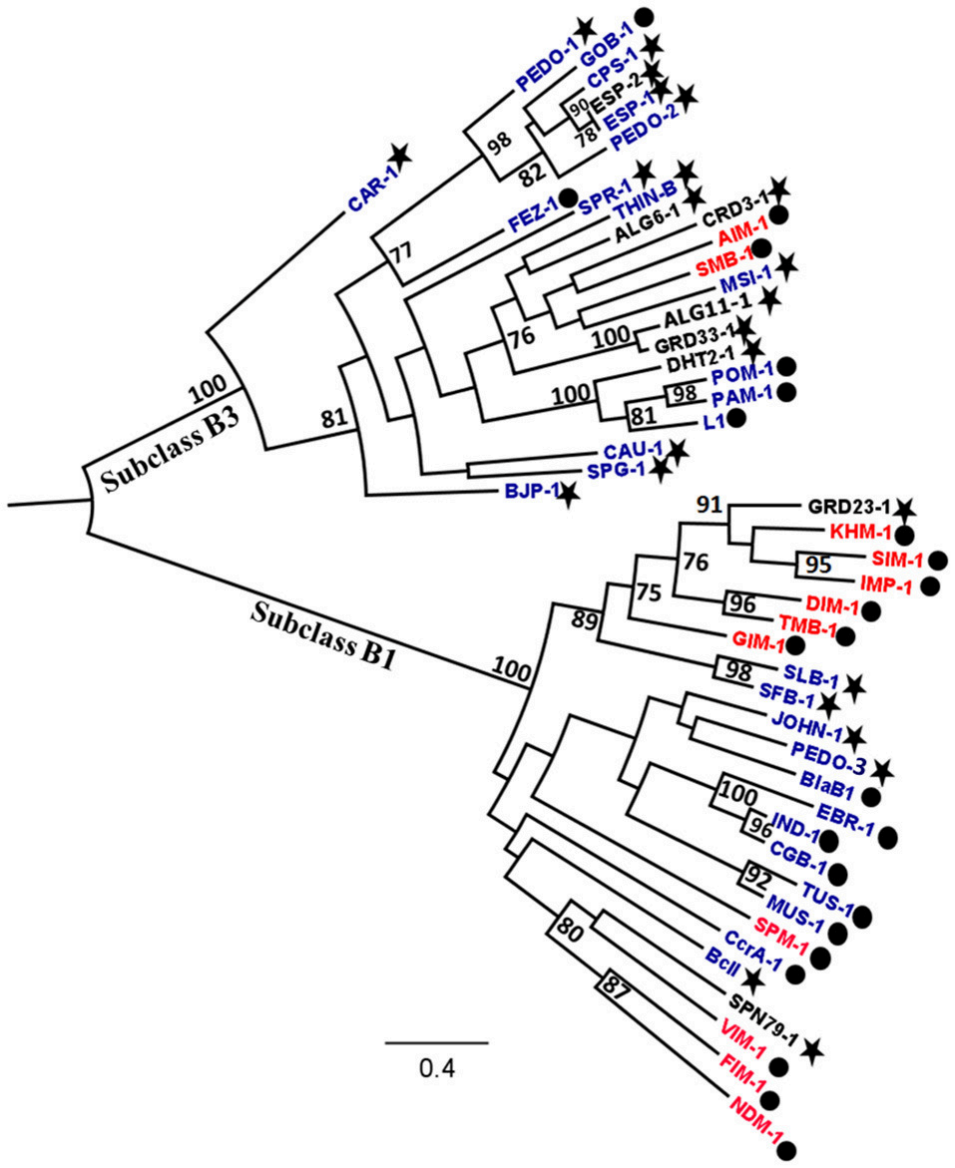
**Mid-point rooted phylogenetic tree of subclass B1 and B3 metallo-ß-lactamases (MBLs)**. The evolutionary distances are expressed in unit of amino acid substitution per site. Bootstrapping support using 1000 replicates was applied and is indicated on branches where values are >70%. The new MBLs detected in this study are indicated in black while previously described acquired and resident MBLs are written in red and blue colors, respectively. The MBLs detected in clinical and environmental bacteria are indicated by a dot and a star, respectively.

Among the 94 carbapenemase-positive colonies, nine recombinant clones originating from six soil samples were shown to contain a unique insert (Table 1). All the inserts were genes encoding for MBLs, including two subclass B1 (GRD23-1 and SPN79-1) and seven subclass B3 (CRD3-1, PEDO-1, GRD33-1, ESP-2, ALG6-1, ALG11-1, and DHT2-1). The MBLs identified in three of the libraries by direct plating on selective LB agar corresponded to those detected after overnight enrichment of the same libraries. However, four MBLs (ALG6-1, ALG11-1, DHT2-1, and SPN79-1) were only detected by enrichment. PEDO-1and ESP-2 were identical or closely related to MBLs detected in the previous study by selective culture of soil (100% and 99.7% identity, respectively) (Gudeta et al., 2016a). The seven remaining enzymes were regarded as new MBLs since they showed only 33–59% amino acid identity to known carbapenem-hydrolyzing MBLs (Table 3). All known zinc-binding amino acids in subclasses B1 and B3 enzymes were conserved (Figure 1). The mid-point rooted phylogenetic tree confirmed that the new MBLs belonged to subclasses B1and B3, as supported by high bootstrap values (100%). Three of them (CRD3-1, SPN79-1, and GRD23-1) shared a common ancestor with transferrable MBLs but none was closely related to the main MBLs found in pathogenic bacteria. Although phylogenetic analysis showed a likely evolutionary relationship between SPN79-1 and VIM-1 (Figure 2), SPN79-1 displayed low amino acid identity (<40%) to all VIM-types.

**Table 3:** **Predicted bacterial host and closest homologs of the nine metallo-β-lactamases (MBLs) detected in soil microbiota by functional metagenomics**.

**MBL**	**Predicted bacterial host (genus/phylum)**	**Closest homolog (Accession no.)**	**Bacterial host of MBL homolog**	**Amino acid identity (%)**	**Genetic location**
GRD23-1	*Teredinibacter*/Proteobacteria	KHM-1 (BAH16555.1)	Unknown	55	Plasmid
GRD33-1	*Gemmatimonas*/Gemmatimonatedes	MBL-1^a^ (AIA10668.1)	Unknown	82	Unknown
CRD3-1	*Erythrobacter*/Proteobacteria	AIM-1 (CAQ53840.1)	Unknown	43	Chromosome
DHT2-1	*Burkholderia*/Proteobacteria	L1 (ABO60992.1)	*Stenotrophomonas maltophilia*	59	Chromosome
ALG6-1	*Variovorax*/Proteobacteria	THIN-B (CAC33832.1)	*Janthinobacterium lividum*	52	Chromosome
ALG11-1	*Burkholderia*/Proteobacteria	MBL-1^a^ (AIA10668.1)	Unknown	86	Unknown
SPN79-1	*Phenylobacterium*/Proteobacteria	MBL-2^b^ (KJ694724.1)	Unknown	85	Unknown
PEDO-1	Unidentified^c^/Bacteroidetes	PEDO-1 (KP109677.1)	*Pedobacter roseus*	100	Chromosome
ESP-2	*Pedobacter*/Bacteroidetes	ESP-1 (KP109681.1)	*Epilithonimonas tenax*	99.7	Chromosome

a *Presumptive MBL discovered by functional metagenomics and wrongly annotated as L1 ß-lactamase (Forsberg et al., 2014) despite low amino acid identity (39%) to L1*.

b *Presumptive MBL discovered by functional metagenomics and wrongly annotated as VIM-type ß-lactamase (Forsberg et al., 2014) despite low amino acid identity (<40%) to VIM*.

c *A likely host genus could not be determined by RAIphy*.

### β-lactam susceptibility and functional characterization of MBL-producing *E. coli* clones

Expression of the nine MBLs recovered from soil microbiota increased the MIC values of ampicillin and cefoxitin above the clinical resistance breakpoints in *E. coli* TOP10. The MICs of 1st and 3rd generation cephalosporins were increased according to various enzyme- and compound-dependent profiles (Table 4). None of the MBL-producing clones exhibited decreased susceptibility to cefepime, a 4th generation cephalosporin. As for carbapenems, expression of all MBLs determined a 2- to 16-fold increase in the MIC of imipenem compared to the wild type strain, and all except SPN79-1 resulted in 2- to 64-fold higher MICs of meropenem (Table 4). However, for none of the MBLs, the MIC of imipenem was increased above the CLSI clinical resistance breakpoint (R ≥ 4 μg/ml), and clinical resistance to meropenem was only achieved through expression of ALG11-1, CRD3-1, and GRD23-1.

**Table 4:** **Imipenem hydrolytic activity and minimum inhibitory concentration (MIC) of selected ß-lactams in *Escherichia coli* TOP10 expressing nine distinct metallo-ß-lactamases (MBLs) recovered from soil microbiota by functional metagenomics**.

***E. coli* TOP10 strain**	**IPM- hydrolyzing activity^a^**	**MIC (μg/ml)**										
		**AMP**	**FOX**	**CFZ**	**CEF**	**CTX**	**CAZ**	**POD**	**CRO**	**FEP**	**IPM**	**MEM**
Wild type	–	≤ 8	8	≤ 8	≤ 8	0.01	0.06	0.5	0.01	0.125	0.016	0.125
p(GRD23-1)	63 ± 9	>16	>64	>16	>16	16	8	>32	128	0.125	0.25	8
p(GRD33-1)	340 ± 50	>16	64	16	>16	0.5	0.5	4	2	0.125	0.063	0.5
p(CRD3-1)	1120 ± 125	>16	>64	>16	>16	4	16	32	16	0.125	0.063	4
p(PEDO-1)	190 ± 16	>16	>64	16	>16	1	16	4	0.01	0.125	0.063	0.5
p(ESP-2)	43 ± 5	>16	>64	≤ 8	>16	0.02	2	2	0.01	0.125	0.063	0.5
p(SPN79-1)	37 ± 3	>16	16	≤ 8	>16	0.01	8	4	0.06	0.125	0.063	0.125
p(DHT1-1)	160 ± 10	>16	64	≤ 8	>16	0.01	1	2	2	0.125	0.063	1
p(ALG6-1)	7 ± 2	>16	>64	>16	>16	16	128	16	32	0.125	0.031	0.25
p(ALG11-1)	620 ± 37	>16	>64	>16	>16	0.5	0.1	2	2	0.125	0.125	4

*AMP, ampicillin; FOX, cefoxitin; CFZ, cefazolin; CEF, cephalothin; CTX, cefotaxime; CAZ, ceftazidime; POD, cefpodoxime; CRO ceftriaxone; FEP, cefepime; IPM, imipenem; MEM, meropenem*.

a *IPM-hydrolyzing activity measured in bacterial crude extracts and expressed in nmol of imipenem hydrolyzed/minute·mg of total protein*.

### Carbapenemase activity of MBLs

The enzymatic assays showed a variable amount of imipenem-hydrolyzing activity in the various crude extracts (Table 4). Crude extract from the CRD3-1-producing strain showed the highest specific activity (1120 ± 125 nmol/min·mg of protein). Notable imipenem-hydrolyzing activities were also detected in crude extracts of the strains producing ALG11-1, GRD33-1, PEDO-1, DHT2-1, SPN79-1, ESP-2, and GRD23-1 (37–620 nmol/min·mg of protein). Only the crude extract from the ALG6-1-producing strain exhibited low imipenem-hydrolyzing activity, which was consistent with the low imipenem MIC observed in this strain. It is interesting to note that the production of ALG6-1 in *E. coli* yielded a phenotype notably different from that mediated by the other MBLs, with high-level resistance to ceftazidime and relatively limited activity on carbapenems (Table 4). Thus, the substrate profile of ALG6-1 was unusual, resembling that of CAR-1 from *Erwinia carotovora* (Stoczko et al., 2008).

### Culture-based method vs. functional metagenomics

We compared the carbapenemase yield obtained by functional metagenomics to that obtained by antibiotic selective culture of the ten soil samples analyzed in this study (Gudeta et al., 2016a). Three samples (ID 6, 7, and 10) did not yield any carbapenemase-encoding gene by both methods. The metagenomics approach detected at least one MBL gene from four samples that did not yield any carbapenemase producers by selective culture (Table 1). On the contrary, in one sample (ID 4), carbapenemase-producing bacteria were detected by culture but no carbapenemase-encoding genes were detected by functional metagenomics. Finally, in two samples (ID 3 and 23) both methods allowed detection of carbapenemase-encoding genes but different types were detected depending on the method used (Table 1). Three soil samples yielded simultaneously two MBL-encoding genes by functional metagenomics and one of these samples (ID 23) was also the only soil yielding more than one (*n* = 4) MBL producers by antibiotic selective culture.

### Prediction of bacterial hosts

According to prediction analysis using RAIphy, six enzymes (CRD3-1, GRD23-1, DHT2-1, SPN79-1, ALG6-1, and ALG11-1) originated from *Proteobacteria*, two (PEDO-1 and ESP-2) from *Bacteroidetes*, and one (GRD33-1) from *Gemmatimonadetes*. The two former phyla (*Proteobacteria* and *Bacteroidetes*) were also represented among the MBL producers isolated by selective culture in the previous study (Gudeta et al., 2016a), whereas the third one (*Gemmatimonadetes*) was only detected by functional metagenomics. The likely genus from which *bla*_PEDO−1_ originated could not be determined.

## Discussion

Nine carbapenem-hydrolyzing MBLs, including seven novel enzymes, were identified in six out of ten soil samples using a functional metagenomics approach. All these enzymes showed hydrolytic activity toward imipenem (as measured in bacterial crude extracts) and increased the MICs of different ß-lactam agents in *E. coli*, thus confirming their functionality. Based on sequence and phylogenetic analyses, two enzymes belonged to subclass B1, and the remaining seven belonged to B3. Previous studies have highlighted the relative abundance of subclass B3 enzymes in the soil resistome (Rossolini et al., 2001; Docquier et al., 2002; Stoczko et al., 2006; Allen et al., 2009; Gibson et al., 2015). However, carbapenemase activity was not examined in presumptive MBLs reported by previous functional metagenomics studies (Allen et al., 2009; Gibson et al., 2015). Neither subclass B2 nor serine-based (class A and D) enzymes were detected. The lack of subclass B2 enzymes may be due to the screening approach used since amoxicillin is a poor substrate for this MBL subclass. Differently from zinc-based carbapenemases, serine-based carbapenemases have mainly been reported in aquatic bacterial isolates (Henriques et al., 2004; Poirel et al., 2004; Aubron et al., 2005; Girlich et al., 2010; Gudeta et al., 2016b), suggesting that yet unknown ecological factors may play a role in the distribution of these two groups of enzymes in nature. Interestingly, none of the MBLs detected in soil was closely related to common MBLs in clinical bacteria. It will be interesting to see if any of these MBLs will emerge in clinical bacteria in the future.

The new MBLs described in this study showed significant sequence heterogeneity in comparison with previously described MBLs (Table 3). Our phylogenetic analysis shows that within the three subclasses B1 and B3 MBLs do not cluster according to the origin (clinical vs. environmental) of the bacterial hosts (Figure 2), underlining a huge diversity within this family of enzymes. Although transferrable MBLs mainly belong to subclass B1 (Cornaglia et al., 2007; Nordmann et al., 2012), transfer of subclass B3 MBLs such as AIM-1 and SMB-1 from environmental bacteria to human pathogens has been hypothesized (Wachino et al., 2011; Yong et al., 2012). Most of the MBLs identified in soil microbiota by functional metagenomics were predicted to originate from *Proteobacteria*, which comprise the majority of human pathogenic bacterial species. This taxonomic relationship may ease acquisition of new MBL-encoding genes from soil microbiota by pathogenic bacteria.

When expressed in *E. coli*, the MBLs from soil microbiota conferred resistance to different classes of β-lactams, including penicillins and several cephalosporins. None of these enzymes affected the MIC of cefepime, which is in line with the poor catalytic efficiency of MBLs for this substrate (Bellais et al., 2002; Naas et al., 2003; Stoczko et al., 2006). Heterologous expression resulted in higher MICs of carbapenems (Table 4), even though in most cases did not confer resistance according to the CLSI clinical breakpoint (*R* ≥ 4 μg/ml). Other authors have reported low-level resistance to carbapenems as a result of heterologous MBL expression in laboratory *E. coli* strains, regardless if the expressed MBL originated from environmental (Rossolini et al., 2001; Docquier et al., 2002; Stoczko et al., 2006; Gudeta et al., 2016a) or clinical bacteria (Lauretti et al., 1999; Poirel et al., 2000; Gibb et al., 2002; Yong et al., 2012). This apparent incongruity may be explained by the very high permeability of the *E. coli* outer membrane to carbapenems, which results in higher intracellular drug concentrations despite of MBL activity (Matsumura et al., 1999).

The discovery of new MBL-encoding genes from samples that did not yield MBL producers by culture suggests that those genes may originate from the unculturable fraction of the soil microbiota or from bacteria non-culturable under the conditions used in the previous study (Gudeta et al., 2016a). On the other hand, none of the MBLs previously identified by culture were re-detected by functional metagenomics in the 10 samples that were analyzed by both methods (Table 1). The lack of detection of these MBLs by functional metagenomics may be due to various factors, including low concentration of culturable MBL-producers in the sample or loss/dilution of DNA during the cloning procedure. Although this study was not designed to compare the occurrence of MBLs in agricultural and grassland soil, it is interesting to notice that MBLs were detected more frequently and displayed higher diversity in grassland samples than in agricultural samples irrespective of the method used (Table 1).

## Conclusions

Even though the study failed in detecting any close ancestors of the main carbapenemases observed in clinical bacteria, it has improved the current knowledge of the diversity of MBLs in soil microbiota by describing seven new enzymes belonging to subclasses B1 and B3. All these MBLs were functional in *E. coli*, resulting in detectable imipenem-hydrolyzing activity and significantly higher MICs of clinically relevant ß-lactams. The MBLs detected by functional metagenomics differed from those detected in same soils samples by selective culture, showing that the two approaches targeted different subpopulations in soil microbiota. This evidence supports the notion that functional metagenomics and culture may be complementary tools for detection of antibiotic resistance determinants in soil.

## Author contributions

DG performed all the bioinformatic analyses and the laboratory work except for the enzyme activity assay by UV-spectrophotometry, which was carried out by the group at the University of Siena (SP, JD, and GR). VB supervised study design and laboratory work at the University of Copenhagen. EW and GA trained DG in metagenomic library construction at the University of Warwick, the UK. LG conceived the idea of the study, raised the necessary funds and contributed to discussion of the results and writing of the manuscript together with all authors.

### Conflict of interest statement

The authors declare that the research was conducted in the absence of any commercial or financial relationships that could be construed as a potential conflict of interest.

## References

[B1] Allen H. K. Moe L. A. Rodbumrer J. Gaarder A. Handelsman J. (2009). Functional metagenomics reveals diverse β-lactamases in a remote Alaskan soil. ISME J. 3, 243–251. 10.1038/ismej.2008.86 18843302

[B2] Ambler R. P. (1980). The structure of β-lactamases. Philos. Trans. R. Soc. Lond. B Biol. Sci. 289, 321–331. 10.1098/rstb.1980.0049 6109327

[B3] Aubron C. Poirel L. Ash R. J. Nordmann P. (2005). Carbapenemase-producing Enterobacteriaceae, U.S. rivers. Emerg. Infect. Dis. 11, 260–264. 10.3201/eid1102.030684 15752444 PMC3320444

[B4] Bebrone C. Delbruck H. Kupper M. B. Schlomer P. Willmann C. Frere J. M.. (2009). The structure of the dizinc subclass B2 metallo-β-lactamase CphA reveals that the second inhibitory zinc ion binds in the histidine site. Antimicrob. Agents Chemother. 53, 4464–4471. 10.1128/AAC.00288-09 19651913 PMC2764157

[B5] Bellais S. Naas T. Nordmann P. (2002). Genetic and biochemical characterization of CGB-1, an Ambler class B carbapenem-hydrolyzing β-lactamase from *Chryseobacterium gleum*. Antimicrob. Agents Chemother. 46, 2791–2796. 10.1128/AAC.46.9.2791-2796.2002 12183230 PMC127440

[B6] Clinical Laboratory Standards Institute (2015). Methods for Dilution Antimicrobial Susceptibility Tests for Bacteria That Grow Aerobically. Wayne, PA: CLSI document M07-A10.

[B7] Cornaglia G. Akova M. Amicosante G. Cantón R. Cauda R. Docquier J. D.. (2007). Metallo-β-lactamases as emerging resistance determinants in gram-negative pathogens: open issues. Int. J. Antimicrob. Agents 29, 380–388. 10.1016/j.ijantimicag.2006.10.008 17223319

[B8] Cornaglia G. Giamarellou H. Rossolini G. M. (2011). Metallo-β-lactamases: a last frontier for β-lactams? Lancet Infect Dis. 11, 381–393. 10.1016/S1473-3099(11)70056-1 21530894

[B9] Darriba D. Taboada G. L. Doallo R. Posada D. (2011). ProtTest 3: fast selection of best-fit models of protein evolution. Bioinformatics 27, 1164–1165. 10.1093/bioinformatics/btr088 21335321 PMC5215816

[B10] Docquier J. D. Pantanella F. Giuliani F. Thaller M. C. Amicosante G. Galleni M.. (2002). CAU-1, a subclass B3 metallo-β-lactamase of low substrate affinity encoded by an ortholog present in the *Caulobacter crescentus* chromosome. Antimicrob. Agents Chemother. 46, 1823–1830. 10.1128/AAC.46.6.1823-1830.2002 12019096 PMC127251

[B11] Dortet L. Bréchard L. Cuzon G. Poirel L. Nordmann P. (2014). Strategy for rapid detection of carbapenemase-producing Enterobacteriaceae. Antimicrob. Agents Chemother. 58, 2441–2445. 10.1128/AAC.01239-13 24468779 PMC4023735

[B12] Fonseca F. Bromley E. H. Saavedra M. J. Correia A. Spencer J. (2011). Crystal structure of *Serratia fonticola* Sfh-I: activation of the nucleophile in mono-zinc metallo-β-lactamases. J. Mol. Biol. 411, 951–959. 10.1016/j.jmb.2011.06.043 21762699

[B13] Forsberg K. J. Patel S. Gibson M. K. Lauber C. L. Knight R. Fierer N.. (2014). Bacterial phylogeny structures soil resistomes across habitats. Nature 509, 612–616. 10.1038/nature13377 24847883 PMC4079543

[B14] Forsberg K. J. Reyes A. Wang B. Selleck E. M. Sommer M. O. Dantas G. (2012). The shared antibiotic resistome of soil bacteria and human pathogens. Science 337, 1107–1111. 10.1126/science.1220761 22936781 PMC4070369

[B15] Galleni M. Lamotte-Brasseur J. Rossolini G. M. Spencer J. Dideberg O. Frere J. M.. (2001). Standard numbering scheme for class B β-lactamases. Antimicrobials. Agents Chemother. 45, 660–663. 10.1128/AAC.45.3.660-663.2001 11181339 PMC90352

[B16] Gibb A. P. Tribuddharat C. Moore R. A. Louie T. J. Krulicki W. Livermore D. M.. (2002). Nosocomial outbreak of carbapenem-resistant *Pseudomonas aeruginosa* with a new *bla*_IMP_ allele, *bla*_IMP−7_. Antimicrob. Agents Chemother. 46, 255–258. 10.1128/AAC.46.1.255-258.2002 11751148 PMC126979

[B17] Gibson M. K. Forsberg K. J. Dantas G. (2015). Improved annotation of antibiotic resistance determinants reveals microbial resistomes cluster by ecology. ISME J. 9, 207–216. 10.1038/ismej.2014.106 25003965 PMC4274418

[B18] Girlich D. Poirel L. Nordmann P. (2010). Novel Ambler class A carbapenem-hydrolyzing β-lactamase from a *Pseudomonas fluorescens* isolate from the Seine river, Paris, France. Antimicrob. Agents Chemother. 54, 328–332. 10.1128/AAC.00961-09 19901091 PMC2798510

[B19] Gudeta D. D. Bortolaia V. Amos G. Wellington E. M. Brandt K. K. Poirel L.. (2016a). The soil microbiota harbors a diversity of carbapenem-hydrolyzing β-lactamases of potential clinical relevance. Antimicrob. Agents Chemother. 60, 151–160. 10.1128/AAC.01424-15 26482314 PMC4704184

[B20] Gudeta D. D. Bortolaia V. Jayol A. Poirel L. Nordmann P. Guardabassi L. (2016b). *Chromobacterium* spp. harbour Ambler class A β-lactamases showing high identity with KPC. J. Antimicrob.Chemother. 71, 1493–1496. 10.1093/jac/dkw020 26892778

[B21] Henriques I. Moura A. Alves A. Saavedra M. J. Correia A. (2004). Molecular characterization of a carbapenem-hydrolyzing class A β-lactamase, SFC-1, from *Serratia fonticola* UTAD54. Antimicrob. Agents Chemother. 48, 2321–2324. 10.1128/AAC.48.6.2321-2324.2004 15155245 PMC415594

[B22] Lauretti L. Riccio M. L. Mazzariol A. Cornaglia G. Amicosante G. Fontana R.. (1999). Cloning and characterization of *bla*_VIM_, a new integron-borne metallo-β-lactamase gene from a *Pseudomonas aeruginosa* clinical isolate. Antimicrob. Agents Chemother. 43, 1584–1590. 10390207 10.1128/aac.43.7.1584 PMC89328

[B23] Matsumura N. Minami S. Watanabe Y. Iyobe S. Mitsuhashi S. (1999). Role of permeability in the activities of β-lactams against gram-negative bacteria which produce a group 3 β-lactamase. Antimicrob. Agents Chemother. 43, 2084–2086. 10428944 10.1128/aac.43.8.2084 PMC89422

[B24] Naas T. Bellais S. Nordmann P. (2003). Molecular and biochemical characterization of a carbapenem-hydrolysing β-lactamase from *Flavobacterium johnsoniae*. J. Antimicrob. Chemother. 51, 267–273. 10.1093/jac/dkg069 12562690

[B25] Nalbantoglu O. U. Way S. F. Hinrichs S. H. Sayood K. (2011). RAIphy: phylogenetic classification of metagenomics samples using iterative refinement of relative abundance index profiles. BMC Bioinform. 12:41 10.1186/1471-2105-12-41 21281493 PMC3038895

[B26] Nesme J. Simonet P. (2015). The soil resistome: a critical review on antibiotic resistance origins, ecology and dissemination potential in telluric bacteria. Environ. Microbiol. 17, 913–930. 10.1111/1462-2920.12631 25286745

[B27] Nordmann P. Dortet L. Poirel L. (2012). Carbapenem resistance in *Enterobacteriaceae*: here is the storm! Trends Mol. Med. 18, 263–272. 10.1016/j.molmed.2012.03.003 22480775

[B28] Nordmann P. Naas T. Poirel L. (2011). Global spread of carbapenemase-producing Enterobacteriaceae. Emerg. Infect Dis. 17, 1791–1798. 10.3201/eid1710.110655 22000347 PMC3310682

[B29] Poirel L. Heritier C. Nordmann P. (2004). Chromosome-encoded Ambler class D β-lactamase of *Shewanella oneidensis* as a progenitor of carbapenem-hydrolyzing oxacillinase. Antimicrob. Agents Chemother. 48, 348–351. 10.1128/AAC.48.1.348-351.2004 14693565 PMC310178

[B30] Poirel L. Naas T. Nicolas D. Collet L. Bellais S. Cavallo J. D.. (2000). Characterization of VIM-2, a carbapenem-hydrolyzing metallo-β-lactamase and its plasmid- and integron-borne gene from a *Pseudomonas aeruginosa* clinical isolate in France. Antimicrob. Agents Chemother. 44, 891–897. 10.1128/AAC.44.4.891-897.2000 10722487 PMC89788

[B31] Rossolini G. M. Condemi M. A. Pantanella F. Docquier J. D. Amicosante G. Thaller M. C. (2001). Metallo-β-lactamase producers in environmental microbiota: new molecular class B enzyme in *Janthinobacterium lividum*. Antimicrob. Agents Chemother. 45, 837–844. 10.1128/AAC.45.3.837-844.2001 11181369 PMC90382

[B32] Saavedra M. J. Peixe L. Sousa J. C. Henriques I. Alves A. Correia A. (2003). Sfh-I, a subclass B2 metallo-β-lactamase from a *Serratia fonticola* environmental isolate. Antimicrob. Agents Chemother. 47, 2330–2333. 10.1128/AAC.47.7.2330-2333.2003 12821491 PMC161872

[B33] Silvestro D. Michalak I. (2012). raxmlGUI: a graphical front-end for RAxML. Organ. Divers. Evol. 12, 335–337. 10.1007/s13127-011-0056-0

[B34] Sommer M. O. Dantas G. Church G. M. (2009). Functional characterization of the antibiotic resistance reservoir in the human microflora. Science 325, 1128–1131. 10.1126/science.1176950 19713526 PMC4720503

[B35] Stoczko M. Frère J.-M. Rossolini G. M. Docquier J.-D. (2006). Postgenomic scan of metallo-β-lactamase homologues in Rhizobacteria: identification and characterization of BJP-1, a subclass B3 ortholog from *Bradyrhizobium japonicum*. Antimicrob. Agents Chemother. 50, 1973–1981. 10.1128/AAC.01551-05 16723554 PMC1479130

[B36] Stoczko M. Frère J.-M. Rossolini G. M. Docquier J.-D. (2008). Functional diversity among metallo-ß-lactamases: characterization of the CAR-1 enzyme of *Erwinia carotovora*. Antimicrob. Agents Chemother. 52, 2473–2479. 10.1128/AAC.01062-07 18443127 PMC2443915

[B37] Tahlan K. Jensen S. E. (2013). Origins of the β-lactam rings in natural products. J. Antibiot. 66, 401–410. 10.1038/ja.2013.24 23531986

[B38] Wachino J. Yoshida H. Yamane K. Suzuki S. Matsui M. Yamagishi T.. (2011). SMB-1, a novel subclass B3 metallo-β-lactamase, associated with ISCR1 and a class 1 integron, from a carbapenem-resistant *Serratia marcescens* clinical isolate. Antimicrob. Agents Chemother. 55, 5143–5149. 10.1128/AAC.05045-11 21876060 PMC3195065

[B39] Walsh T. R. Toleman M. A. Poirel L. Nordmann P. (2005). Metallo-β-lactamases: the quiet before the storm? Clin. Microbiol. Rev. 18, 306–325. 10.1128/CMR.18.2.306-325.2005 15831827 PMC1082798

[B40] Yong D. Toleman M. A. Bell J. Ritchie B. Pratt R. Ryley H.. (2012). Genetic and biochemical characterization of an acquired subgroup B3 metallo-β-lactamase gene, *bla*_AIM−1_, and its unique genetic context in *Pseudomonas aeruginosa* from Australia. Antimicrob. Agents Chemother. 56, 6154–6159. 10.1128/AAC.05654-11 22985886 PMC3497169

